# Unveiling errors in soil microbial community sequencing: a case for reference soils and improved diagnostics for nanopore sequencing

**DOI:** 10.1038/s42003-024-06594-8

**Published:** 2024-07-28

**Authors:** Daniel K. Manter, Catherine L. Reardon, Amanda J. Ashworth, Abasiofiok M. Ibekwe, R. Michael Lehman, Jude E. Maul, Daniel N. Miller, Timothy Creed, Patrick M. Ewing, Stanley Park, Thomas F. Ducey, Heather L. Tyler, Kristen S. Veum, Sharon L. Weyers, David B. Knaebel

**Affiliations:** 1 Soil Management and Sugar Beet Research, United States Department of Agriculture Agricultural Research Service (USDA-ARS), Fort Collins, CO USA; 2 Soil and Water Conservation Research Unit, USDA-ARS, Adams, OR USA; 3 Poultry Production and Product Safety Research Unit, USDA-ARS, Fayetteville, AR USA; 4 Water Efficiency and Salinity Research Unit, USDA-ARS, Riverside, CA USA; 5 North Central Agricultural Research Laboratory, USDA-ARS, Brookings, SD USA; 6 Sustainable Agricultural Systems Laboratory, USDA-ARS, Beltsville, MD USA; 7 Agroecosystem Management Research Unit, USDA-ARS, Lincoln, NE USA; 8 Food Systems Research Unit, USDA-ARS, Burlington, VT USA; 9 Coastal Plains Soil, Water and Plant Research Center, USDA-ARS, Florence, SC USA; 10 Crop Production Systems Research Unit, USDA-ARS, Stoneville, MS USA; 11 Cropping Systems and Water Quality Research Unit, USDA-ARS, Columbia, MO USA; 12 Soil Management Research Unit, USDA-ARS, Morris, MN USA; 13 Federal government retiree, Fayetteville, NY USA

**Keywords:** Molecular biology, Computational biology and bioinformatics

## Abstract

The sequencing platform and workflow strongly influence microbial community analyses through potential errors at each step. Effective diagnostics and experimental controls are needed to validate data and improve reproducibility. This cross-laboratory study evaluates sources of variability and error at three main steps of a standardized amplicon sequencing workflow (DNA extraction, polymerase chain reaction [PCR], and sequencing) using Oxford Nanopore MinION to analyze agricultural soils and a simple mock community. Variability in sequence results occurs at each step in the workflow with PCR errors and differences in library size greatly influencing diversity estimates. Common bioinformatic diagnostics and the mock community are ineffective at detecting PCR abnormalities. This work outlines several diagnostic checks and techniques to account for sequencing depth and ensure accuracy and reproducibility in soil community analyses. These diagnostics and the inclusion of a reference soil can help ensure data validity and facilitate the comparison of multiple sequencing runs within and between laboratories.

## Abstract (web summary)

A soil bacterial amplicon sequencing study using the Nanopore MinION platform suggests that common diagnostics are insufficient to detect irregular community results and recommends further diagnostics and the use of a reference soil as a mock community.

## Introduction

The soil microbiome is crucial to soil sustainability and a driver for processes of decomposition, biogeochemical cycling, and physical structuring of soil imparting both direct and indirect effects on the soil environment^1^. However, the response of soil biota to environmental changes, either short-term or long-term, has motivated research to evaluate microbial assemblages as indicators of perturbation, management, or soil health outcomes^2–4^. There is growing interest in the identification of important taxonomic and functional abundance shifts related to soil processes^2^; therefore, amplicon or marker-gene sequencing have become standard tools in soil science^5^. These methods have greatly advanced our understanding of microbial community dynamics and biological interactions within the soil environment; however, the spatiotemporal complexity, heterogeneity, and high biological diversity of soil microbial communities present unique challenges to research^2,3,6^. As soil community analyses become more widely applied, the influence of methodology and reproducibility between laboratories and sequencing runs are increasingly more apparent^7^ and necessitate new diagnostic tools and/or reference standards.

Molecular analyses to assess soil biological diversity include many steps in the workflow, each of which may influence the final result^7^. Early steps in DNA extraction and lysis procedures affect the downstream results such as recovered DNA concentrations^8–10^, composition^7,8,11–13^, and diversity^7,14^. However, each step including primer selection^13,15,16^, polymerase chain reaction (PCR) protocol^11,17^, and PCR chemistries^12^ also impart variability. Biases introduced by PCR^18^ and/or DNA extraction^7,11^ can outweigh the biases introduced in downstream sequencing steps.

The potential to introduce systemic errors or bias in a sequential workflow with methodological differences makes data harmonization for meta-analysis of independent study results unfeasible^6^. Additionally, approaches to normalize and compare microbiome data across laboratories are not yet established^13^. Guidelines on the minimum reporting information and quality for amplicon^19^ and marker-gene sequencing^20^ have been developed to support reproducibility, though lab-to-lab variability still remains a challenge to inter-experimental comparisons^12,13,21^. These biases may be further exacerbated by rapidly evolving technology, including novel or discontinued sequencing platforms^16^ and commercial products^10,21^.

Mock or artificial communities are commonly used and recommended as controls to help identify bias or variability and optimize workflows across different biological systems^7,11–13,21,22^. Mock community controls, either available commercially or developed in-house, include mixes of whole cells of a few to tens of species in known ratios^10,23^, genomic DNA with up to as many as 87 genomic strains^24,25^, or DNA fragments such as spike-in sequences^26^. However, mock communities are usually less complex than the target system, particularly for soil ecosystems, and often do not reflect the taxonomic composition, structure, and diversity of natural communities^7,11,22^. Due to these differences, mock communities may not be suitable for identifying errors and/or biases that arise in more complex samples, such as soils.

Trends in agricultural research and molecular microbiology are leaning toward large-scale, long-term studies that are often supported by multiple laboratories or multiple sequence runs. These types of large-scale studies are likely at higher risk of variability or bias; however, the extent of this effect is largely unknown. Cross-laboratory studies and complex mock communities are needed to evaluate biases associated with lab effect including sources of variability and error during each of the major workflow steps. Unlike other science fields where reference materials are widely available for method validation, quality control, and estimation of variability or error^27^, no such mock or reference material exists for soil molecular analyses. Highly characterized reference soils are available for nutrient analyses; however, these reference soils are not characterized or stored in a way that would make them usable for DNA analyses. Standards of sufficient complexity, either mock communities or reference soils, that could be used for molecular comparisons of soil communities are not yet available.

New opportunities for cross-laboratory studies to evaluate sequencing bias and variability have arisen with the introduction of relatively low-cost DNA sequencing technology that supports in-house analyses. This study employed a multi-laboratory 16S rRNA amplicon sequencing analysis to: (i) develop a standardized protocol for amplicon sequencing with the Oxford Nanopore MinION system; (ii) test its reproducibility across individual laboratories; and (iii) develop bioinformatic diagnostics to identify library inconsistencies and highlight the use of reference soils to assist with identifying problematic sequence runs. Of the six participating laboratories, one produced aberrant sequence runs that could only be identified through comparison to others. This unique dataset provided the opportunity to develop diagnostic tools and quality controls in concert with both simple (commercial mock community of genomic DNA from 9 bacterial species) and complex (soil) communities for improved reproducibility and the identification of aberrant sequence runs and sources of error.

## Results

**Fig. 1:**
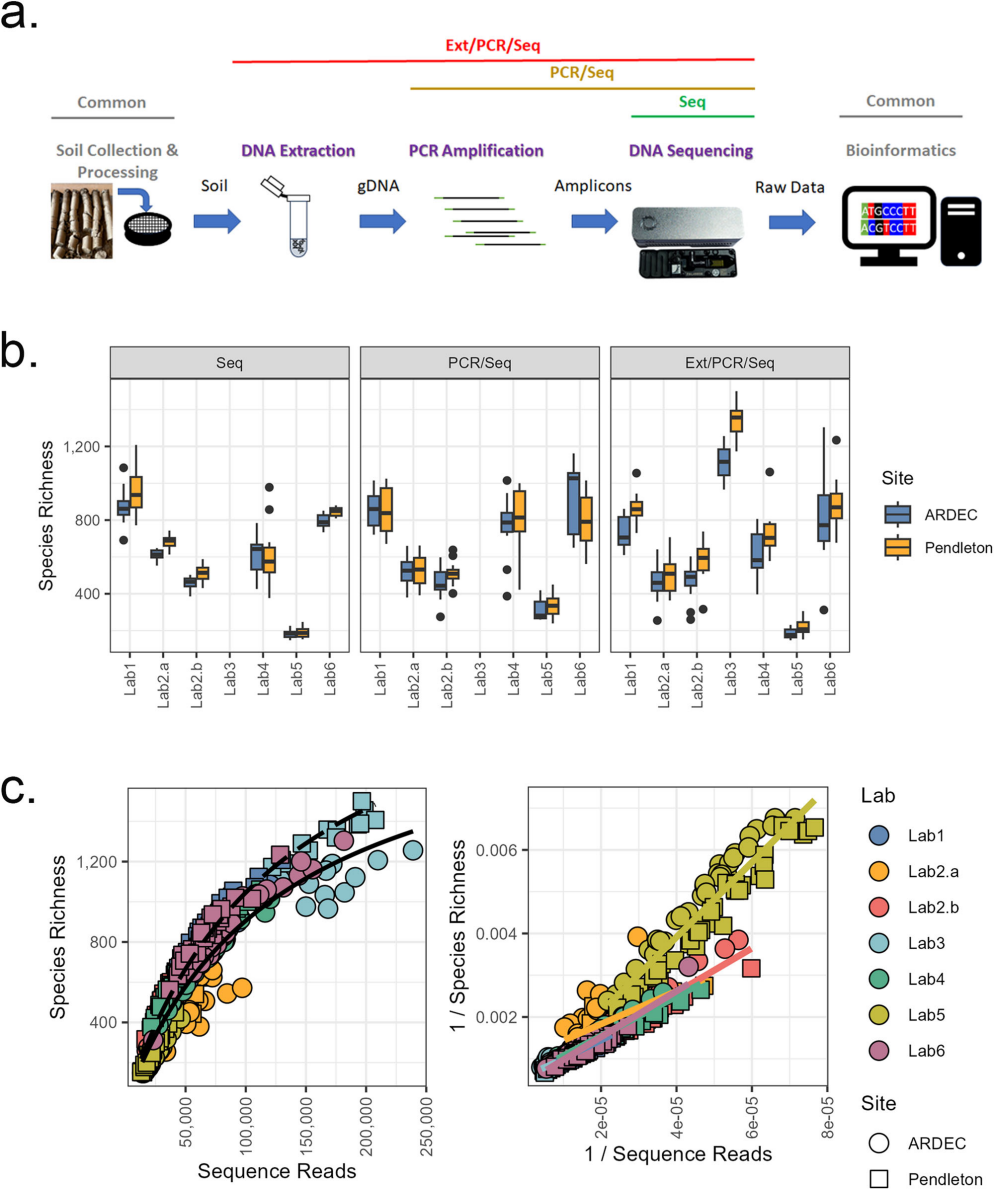
Experimental design and species richness for community sequence data generated with varying levels of workflow autonomy. **a** The typical steps (e.g. DNA extraction, PCR amplification, and DNA sequencing) involved in generating soil microbial community sequence data were evaluated as sources of variability. The bars above the steps denote the processes performed by secondary labs to generate each of the three libraries (Ext/PCR/Seq, PCR/Seq, and Seq) that were pooled prior to sequencing on the MinION platform (ONT). **b** Species richness for two soil sites (*n* = 12) generated in each sequencing run (Lab1, Lab2.a, etc.) and within each of the three pooled libraries. Data were rarefied to 12,000 reads prior to calculating richness estimates. Each box depicts the interquartile range (IQR), where the bottom of the bar is the 1st quartile (Q1), the middle bar is the 2nd quartile or median, and the top of the bar is the 3rd quartile (Q3). Whiskers are calculated as 1.5 × IQR above Q3 or 1.5 × IQR below Q1 and points outside this range are outliers. **c** The relationship between the original number of sequence reads and observed species richness for each sample. Left: Michaelis–Menten plot where points are coloured by sequence run and equations were fit separately for each site (ARDEC, solid line; Pendleton, dashed line). Right: Lineweaver–Burk plot where points and lines are coloured by laboratory and equations were fit separately for each sequence run. All images in this figure are original artwork by the authors.

In this study, six laboratories sequenced two agricultural soils and a simple mock community with increasing levels of autonomy (e.g. self-completion) at each of the three main workflow steps: DNA extraction, PCR amplification, and library preparation and pooling. To achieve varying levels of autonomy, each laboratory received samples from one of two primary labs that were at varying stages of preparation: (i) soils which required DNA extraction, PCR, and sequencing (Ext/PCR/Seq), (ii) soil DNA extracts that required only PCR and sequencing (PCR/Seq), and (iii) a DNA library prepared by the primary lab which only needed to be sequenced (Seq) (Fig. 1). The secondary lab was responsible for completion of the remaining steps in the workflow for each library and conducting a single MinION sequencing run that contained all three libraries. This design allowed for the evaluation of (i) the influence of overall sequencing run characteristics (e.g. library size and quality); (ii) within-laboratory variability; and (iii) between-laboratory variability. In addition, the different levels of autonomy were used to evaluate the steps in the workflow where errors/biases were most likely introduced. For all analyses, sequence run refers to the single MinION run performed in each laboratory, library refers to the varying preparation of samples within a library (PCR, PCR/Seq, and Ext/PCR/Seq), and site (ARDEC/Pendleton) is the source of the original soil sample.

### Sequence run sizes and QC

A total of 5–12 M reads were obtained from each MinION sequence run that included the three pooled libraries produced with increasing levels of workflow autonomy from Seq > PCR/Seq > Ext/PCR Seq (Supplementary Table 1 and Fig. 1a). On average, 42.4% of the sequence reads were removed during the de-multiplexing step, an additional 6.8% removed during the QC-quality step, and a final removal of less than 1.0% of the sequences during the final QC-length step. The final number of high-quality sequence reads ranged from 1.8 M to 5.8 M reads per sequencing run.

The number of reads per soil sample trended similarly to the total number of quality-filtered reads per sequence run except for Lab3 which sequenced only one of the three libraries. Lab3 had more sequence reads per sample than any other library for soil (130–660% more reads than other labs), MOCK (80–650% more reads than other labs), and the controls for DNA extraction (ExtH2O) and PCR1 (PCR1H2O). Lab5, which had the greatest removal of sequences during the demultiplex and QC-quality steps, had the lowest number of reads per sample for both soils and MOCK. The number of reads per sample for the ExtH2O and PCR1H2O controls was less than 0.4% of the total quality-filtered reads per sequence run except for Lab3. Although the mock community was not included in the Seq library from primary Lab1, the percent MOCK reads per total quality-filtered reads was similar between the sequence runs of the two primary labs (primary Lab1, 1.9–2% MOCK reads; primary Lab4, 1.6–2.1% MOCK reads) with the exclusion of data from Lab3 that included only the Ext/PCR/Seq library.

### Mock community

The mock community showed little deviation in community composition across all sequence runs (Supplementary Fig. 1). Each sequence run captured all eight genera of ZymoBIOMICS Microbial Community Standard in the MOCK sample with less than 0.1% of the community from contaminants. All samples had Bray–Curtis (BC) similarities to the expected MOCK profile of greater than 0.860 with average (±standard deviation [SD]) BC similarities of 0.890 ± 0.002, 0.878 ± 0.018, and 0.883 ± 0.016 for the Seq, PCR/Seq, and Ext/PCR/Seq, respectively. When comparing between sequence runs, all MOCK samples demonstrated similar profiles with BC similarities above 0.838 regardless of the library preparation. Negative (PCR1H2O and PCR2H2O) and simple mock (MOCK) control results together suggested that all libraries and sequence runs were successfully prepared and executed and would produce similar estimates of microbial community profiles. However, as discussed below, complex soil samples demonstrated increased variability and differences between laboratories.

### Soils—α diversity

Species richness estimates, calculated after rarefying to a common sampling depth, ranged from approximately 200–1200 species per soil sample (Fig. 1b) and were significantly (*P* < 0.001) influenced by sequence run, site, and library. Sequence run had the greatest partial effect size (η^2^) on species richness (*F* = 362.6, η^2^ = 0.80) followed by site (*F* = 17.84, η^2^ = 0.16) and library (*F* = 6.878, η^2^ = 0.04). Despite rarefying, a strong non-linear relationship was observed between the original number of sequence reads and the estimated species richness. Rectangular hyperbola (i.e. Michaelis–Menten) curves of species richness to original sequence reads were fitted separately for each site across all sequence runs and libraries (Fig. 1c). Maximum species richness (±SE) differed between the ARDEC (2093 ± 82) and Pendleton (2412 ± 79) sites. Lineweaver–Burk (LB) plots showed that two of the sequence runs deviated from the others (Fig. 1c). Lab2.a had a significantly different intercept (*P* = 0.002) and Lab5 had a significantly different slope (*P* < 0.001).

### Soils—β diversity

**Fig. 2:**
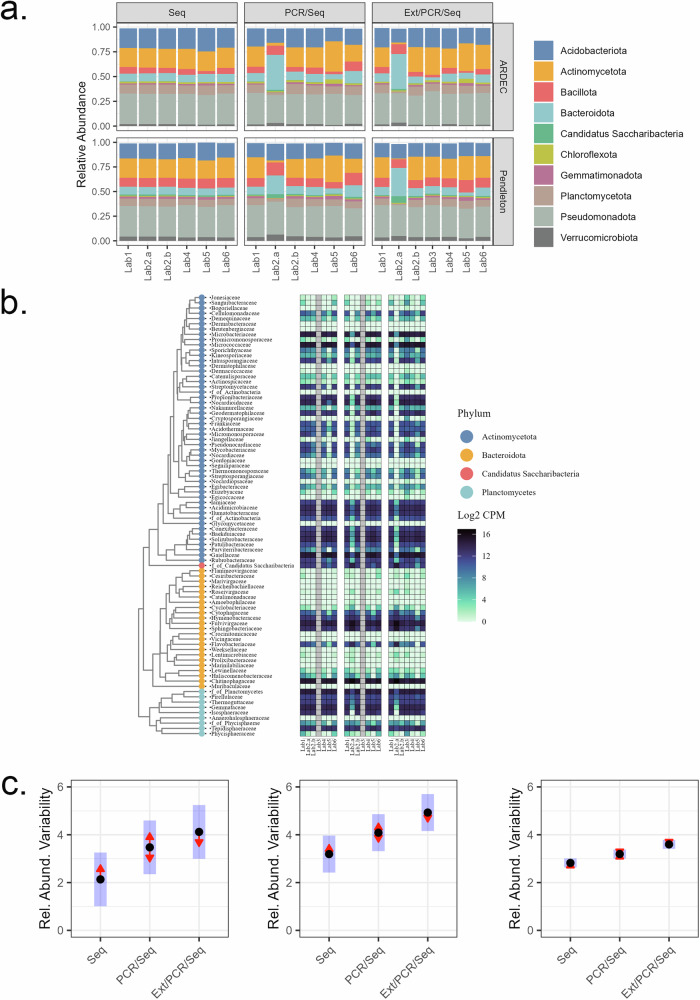
β-diversity of bacterial communities analysed with increasing workflow autonomy. **a** The relative abundance of phyla for agricultural soils collected at two sites (ARDEC, Pendleton) and analysed by six participating laboratories with increasing levels of library process autonomy (Seq < PCR/Seq < Ext/PCR/Seq). Bars indicate each separate sequence run with two runs complete for Lab2 (Lab2.a, first run; Lab2.b, repeat). **b** Family-level taxonomic abundances for four selected bacterial phyla. Each cell is the log2 abundance (counts per million reads) averaged across replicate soil samples for each sequencing run/library combination. **c** Variability in Log2 fold-change (Log2FC) comparing taxonomic abundances (counts per million reads) for each sequencing run at the phylum (left), class (middle), and genus (right) taxonomic levels. Variability was calculated as the range (i.e. abs (max–min)) in Log2FC values comparing each sequencing run with the average of the two primary labs (Lab1 and Lab4) for all of the 26 phyla, 71 classes, and 1019 genera in the entire dataset. Lab3 was not included in the analysis as it did not include the Seq and PCR/Seq libraries. Each point is the average Log2FC range with blue bars indicating the 95% confidence interval. Overlapping red arrowed lines indicate non-significant comparisons (*P* *>* 0.05) based on repeated measures ANOVA and FDR-adjusted p-values.

Similar patterns in the relative abundance of the top 10 phyla were observed between sequence runs except for Lab2.a (Fig. 2a). Compared to all other sequence runs, Lab2.a PCR/Seq and Ext/PCR/Seq library runs had significant shifts in the top phyla, including Bacteroidota, Actinomycetota, Planctomycetota, and Candidatus Saccharibacteria. Three of these phyla showed multiple deviations at the family level supporting phyla-wide systematic bias rather than contamination which was also supported by the PCR negative controls (Fig. 2b and Supplementary Table 1). Although sequencing runs Lab2.a and Lab2.b were performed by the same technical staff, the two runs used different thermocyclers. The change in thermocycler did not appear to be the cause of the bias in Lab2.a since replicate reactions performed on the different thermocyclers but sequenced in the Lab2.b sequence run showed a high similarity of 0.895 ± 0.006 (±SD, *n* = 4). The exact origin of the error in Lab2.a could not be identified nor attributed to contamination or differences in equipment. Across all sequence runs, the level of workflow autonomy increased the variability in taxonomic abundances between sequence runs (*P* < 0.001) (Fig. 2c). Variability was estimated as a range in the Log2FC between the average abundance for each sequence run/library combination as compared to the average for the two primary labs (Lab1 and Lab4). For example, at the genus level, the range in Log2FC (±SE) was 3.2 ± 0.4, 4.1 ± 0.4, and 4.9 ± 0.4 for the Seq, PCR/Seq, and Ext/PCR/Seq libraries, respectively.

All library/sequence run combinations were consistently grouped by site and sequence run based on PCoA distance-based analyses of Hellinger-transformed genera abundances (Supplementary Fig. 2a). Site separated along axis 1, which captured about 45% of the variation, and the sequence run separated along axis 2, which described 20.7% of the variation. Further, variance partitioning showed that 47.7%, 26.6%, and 1.1% of the variance was explained by site, sequence run, and library, respectively. Based on perMANOVA analysis, site was significantly different (*P* = 0.001). In each library, Lab5 was significantly different (*P* < 0.05) from all other sequence runs except for Lab2.b in the two least autonomous libraries (Seq and PCR/Seq). Comparatively, the two most autonomous libraries (PCR/Seq and Ext/PCR/Seq) for Lab2.a were significantly different (*P* = 0.001) from all other sequence runs. Consistent with the α-diversity measurements, the differences between sequence runs appeared to be linked to library size as both axes 1 and 2 exhibited a non-linear relationship with the original number of sequence reads per sample (Supplementary Fig. 2b).

The PCoA analysis of the Morisita distances calculated from untransformed genera abundance data was consistent with the BC distances of Hellinger-transformed data (Supplementary Fig. 3). Similarly, the two axes explained 57.4% (axis 1) and 30.2% (axis 2) of the variation with the two soils separating along axis 1 (perMANOVA analysis, *P* < 0.05). Variance partitioning showed that 62.1%, 22.3%, and 2.6% of the variance was explained by site, sequence run, and library, respectively. Unlike the BC distances, the only sequence run that was significantly different (*P* = 0.001) from the others was Lab2.a in the two most autonomous libraries (PCR/Seq and Ext/PCR/Seq). Morisita distance reduced the effect of sequencing depth on both axes while still maintaining the significant differences between sites (Supplementary Fig. 3b). This suggests that the Morisita distance can mitigate some of the methodological variations in microbial community assessments.

### Within-lab variability

**Fig. 3:**
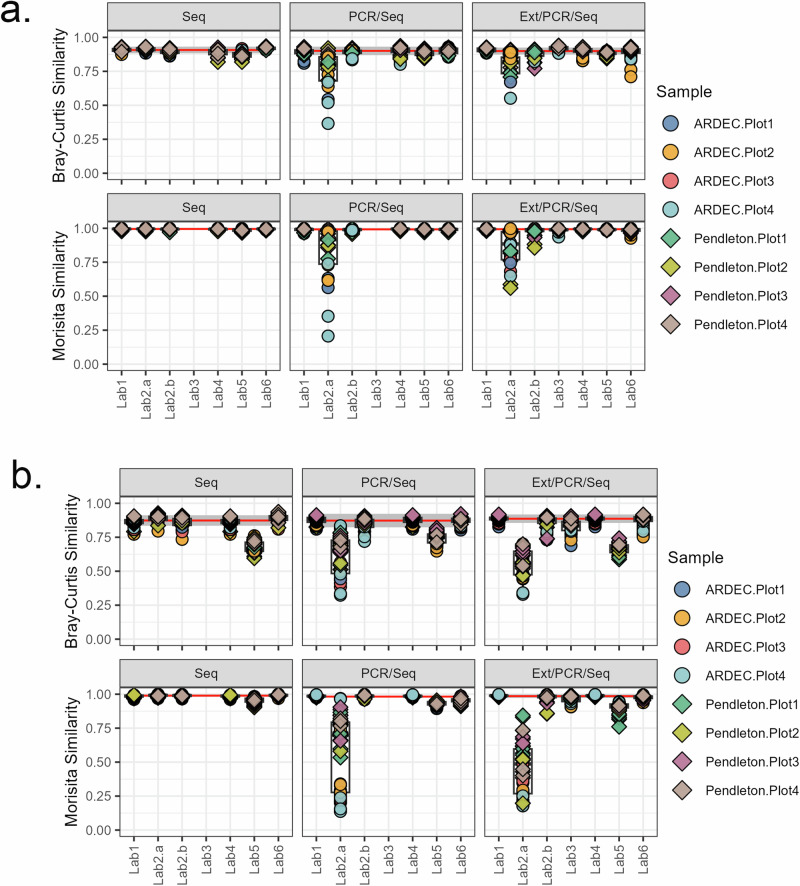
Boxplot of sample variability within each library/sequence run combination. **a** Within-sequencing run variability. Each point is the similarity between replicate soil samples within each sequencing run. For each soil sample, the similarity was calculated from either BC distances calculated from Hellinger-transformed genera relative abundances (top panel) or Morisita distances calculated from untransformed genera relative abundances (bottom panel). **b** Between-sequencing run variability. Each point is the similarity between replicate soil samples compared to a reference soil (Lab1 or Lab4). For each soil sample, the similarity was calculated from either BC distances calculated from Hellinger-transformed genera relative abundances (top panel) or Morisita distances calculated from untransformed genera relative abundances (bottom panel). For both panels, each box depicts the IQR, where the bottom of the bar is the 1st quartile (Q1), the middle bar is the 2nd quartile or median, and the top of the bar is the 3rd quartile (Q3). Whiskers are calculated as 1.5 × IQR above Q3 or 1.5 × IQR below Q1 and points outside this range are outliers. Horizontal red lines are the median for each library and grey bars are the overall median ± IQR for Lab1, Lab2.b, Lab4, and Lab6, collectively.

Extraction and analysis of the soils in triplicate allowed for the development of a sample variability diagnostic for aberrant results based on estimates of within-run variability between sample replicates. Overall, only minor differences in the BC similarities between sample replicates (i.e. subsamples A and B) were observed among libraries with median (±interquartile range [IQR]) values of 0.906 ± 0.030, 0.891 ± 0.044, and 0.896 ± 0.040 for the Seq, PCR/Seq, and Ext/PCR/Seq libraries, respectively (Fig. 3a). When the analysis was conducted for each library/sequence run combination, the PCR/Seq and Ext/PCR/Seq libraries tended to show more variability (i.e. greater IQR’s), particularly for Lab2.a, which had a median (±IQR) of 0.802 ± 0.176 and 0.814 ± 0.120, respectively, than the overall Seq library. The increase in sample variability (i.e. reduced consistency) in Lab2.a starting with the PCR/Seq library suggests that errors arose during the PCR step of the protocol.

### Between-lab variability

To determine whether a complex soil community could serve as a reference in identifying aberrant sequence runs based on low similarity indices, soil sample-level community data were compared between the secondary (sequence run) and corresponding primary lab data (i.e. Lab1 and Lab4) (Fig. 3b). For the Seq libraries, the average median BC similarity was 0.88 for all labs except Lab5. All three libraries of Lab5 exhibited lower BC similarities (e.g. Seq 0.66 ± 0.07, PCR/Seq 0.75 ± 0.06, and Ext/PCR/Seq 0.67 ± 0.06) than most other sequence runs, suggesting that the error arose during the pooling and sequencing step. Notably, Lab5 also had both the highest number of sequences removed during sequence processing (QC quality) and the lowest number of final sequence reads (Supplementary Table 1). Lab2.a exhibited lower BC similarities than most other sequence runs for the two most autonomous libraries (PCR/Seq, 0.65 ± 0.25; Ext/PCR/Seq, 0.56 ± 0.17), supporting that the error arose during PCR.

### Site differences

**Fig. 4:**
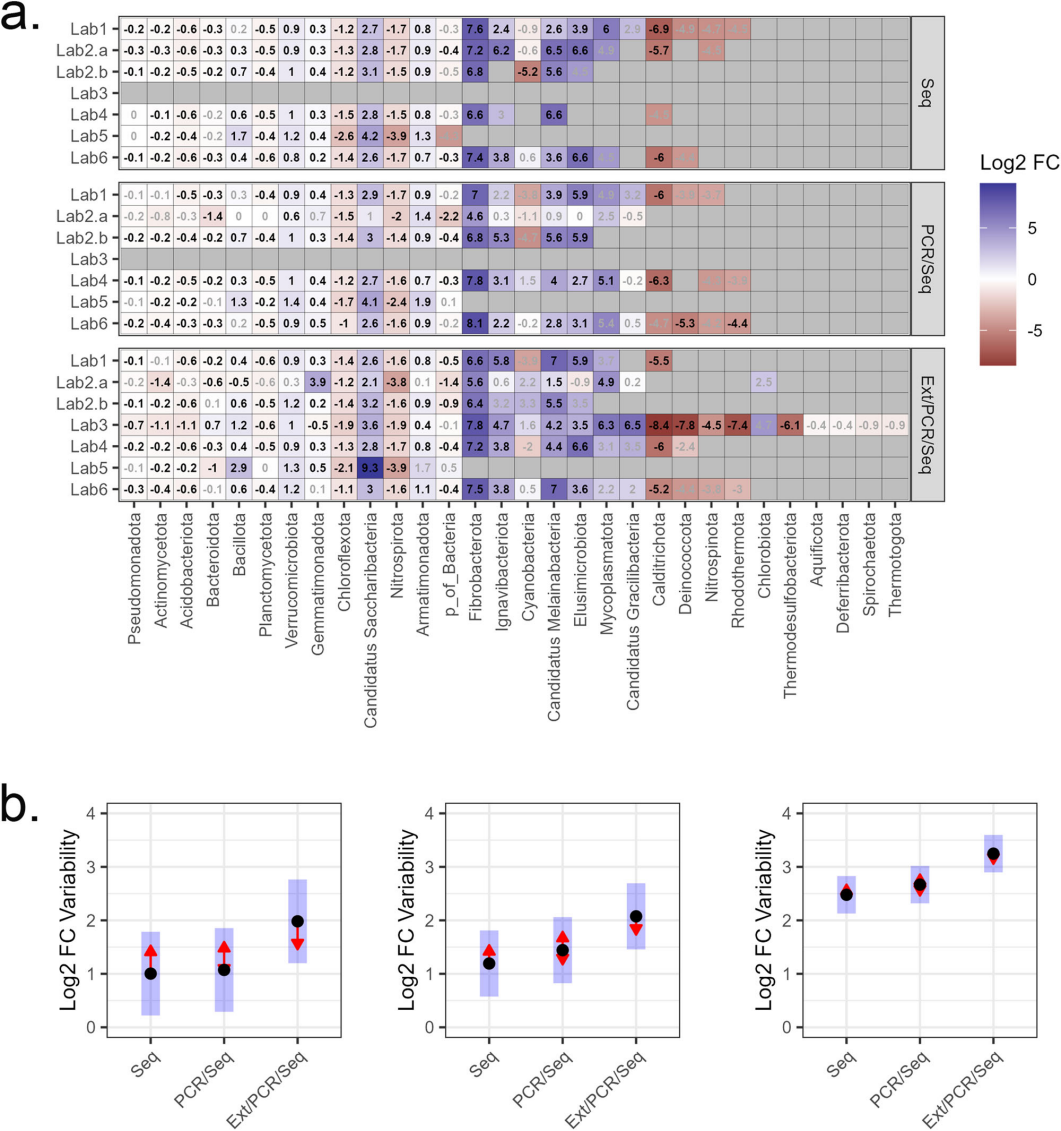
Comparison of Site (soil) effects and sources of variability. **a** Log2FC in the relative abundance of bacterial phyla between soil from the two research sites (ARDEC and Pendleton). The text within each box in the Log2FC with significant (*P* < 0.05) values in black and non-significant in grey. Positive values are enriched at Pendleton and negative values at ARDEC. **b** Variability in Log2FC comparing the two soil sites (ARDEC and Pendleton). Variability was calculated at the phylum (left), class (middle), and genus (right) taxonomic levels as the range (i.e. abs (max–min)) in Log2FC values for each of the 13 phyla, 33 classes, and 161 genera with at least one significant difference between the two sites for all combinations of sequence run and library. Lab3 was not included in the analysis as it did not include the Seq and PCR/Seq libraries. Each point is the average Log2FC range with blue bars indicating the 95% confidence interval. Overlapping red arrowed lines indicate non-significant comparisons (*P* > 0.05) based on repeated measures ANOVA and FDR-adjusted *P*-values.

The number of phyla observed in each sequence run generally trended with sequencing depth ranging from 30 (Lab6) to 13 (Lab5) phyla. Thirteen phyla were shared across each sequence run and library (Fig. 4a). The Seq library had six phyla that differed significantly by Site and across all sequencing runs compared to only four phyla that consistently differed in the PCR/Seq and Ext/PCR/Seq libraries. Omitting Lab5 based on low sequence reads increased the number of shared phyla between sequence runs and the library to 17 with an additional 1–2 phyla that varied significantly by site for each library. In general, statistically significant log2 fold changes (Log2FC) exhibited the same directional patterns across sequence run and library with the exclusion of Bacteroidota, Bacillota, and Gemmatimonadota. For all three taxonomic levels, the variability (i.e. range) of Log2FC values increased with increasing process autonomy (e.g. Seq < PCR/Seq < Ext/PCR/Seq) and finer taxonomic levels (e.g. mean Log2FC variability increased from 2.0 to 3.2 for the phyla and genus levels, respectively) (Fig. 4b).

## Discussion

This study evaluated the reproducibility of a soil community analysis and tested different diagnostics to detect unusual sequence runs. Using a standard protocol to negate workflow effects, six laboratories employed varying levels of autonomy in sequence library preparation with three main entry points of error: DNA extraction, PCR amplification, and sequencing. One of the labs (Lab2.a) produced results with very different taxonomic profiles than the others and two labs generated libraries with relatively low (Lab5) or high (Lab3) sequence reads. Although library size and number of quality-filtered reads identified the labs with the outlier sequence reads, neither the library metrics nor the mock community positive control indicated that Lab2.a was aberrant. With this unique dataset, we identified bioinformatic analyses that could be applied as diagnostics for low-quality or aberrant sequence runs. Throughout, we consider a successful sequence run to be one that generates a soil community profile with high similarity between laboratories.

The introduction of bias or variability within most steps of the microbial community sequencing workflow can impact downstream results (e.g. diversity measures) and potentially skew comparative outcomes and interpretations. In using a single protocol to negate workflow bias, we observed that variability increased with increasing laboratory autonomy in which DNA extraction was a greater source of variability than PCR or the individual MinION sequence device and flow cell.

The Oxford Nanopore MinION platform was selected due to long-read capabilities and relative affordability that supported options for in-house sequencing. Low base-calling accuracy has been a concern for MinION sequencing (accuracy estimated near 95% for MinION compared to 99.9% for Illumina MiSeq^28^) although advancements in accuracy have been made through updated chemistry and flow cells^29^. Error rates of the flow cell (R.9.4.1) and chemistry (SQK-LSK109) used in this study are benchmarked at 7.16% compared to updated products (R10.4.1 flow cell and SQK-LSK114 chemistry) at 3.16%^29^. However, as compared to Illumina MiSeq (V3-4 with DADA2), the MinION protocol used in this study (e.g. full-length 16S rRNA, R9.4.1, SQK-LSK109) is reported to achieve greater similarity to the expected community profile of a mock community for both genus (BC similarity 0.859 vs 0.809) and species (BC similarity 0.852 vs 0.809) level analyses^30^. For comparison, this study demonstrated BC similarities of the mock community to the expected profile of >0.88. Although the R9 flow cells have a higher sequencing error rate than Illumina, this appears to be offset by its ability to generate longer sequence reads resulting in classification rates at least equal to, if not slightly better, than shorter reads on the Illumina MiSeq platform.

Sample heterogeneity and DNA extraction efficiency are potential sources of variability in soil analyses. For this study, we sequenced three replicate sub-samples from a composite sample per plot. Sample heterogeneity was not likely a dominant source of variability in this study because high similarity was observed between the primary labs (Seq library). Similar results are shown with non-soil templates^7,11^. Differences in DNA extraction efficiency between laboratories could potentially impact the reproducibility of sequence results where low concentrations (0.1 ng reaction) of soil DNA template have been associated with greater variability than higher concentrations (5–10 ng per reaction)^31^. Rather than normalizing DNA concentrations, we diluted the soil extracts (i.e. 1:20) to reduce the potential for quantification error and interference from PCR inhibitors. However, extraction efficiency (or template concentration) is also not likely a dominant contributor of variability since PCR template concentration between labs was similar (approximately 6 ng DNA μL^−1^ amplified per reaction). Rather, our data suggest that variability was most likely associated with differences in operator handling (e.g. mixing and pipetting during DNA extraction and other steps) and sequencing error. Regarding the latter, BC similarities for the Seq library (i.e. barcoded library prepared by the primary laboratory) showed similar BC similarities between samples (0.906 ± 0.030) as seen for the ZymoBIOMICS mock communities as compared to their expected profiles (ca. 0.9). We suggest that this represents an approximate maximum threshold similarity between samples that is influenced by the inherent errors due to sequencing platform and bioinformatics, and sampling probabilities that arise from sequencing only a portion of the DNA molecules present in a complex sample.

PCR was the second greatest source of variability as noted for the sample-to-sample comparisons. This step was also the entry point of error for Lab2.a since the Seq library and repeated run (Lab2.b) were consistent with all others. The difference in thermocyclers used for Lab2.a and Lab2.b did not explain the variability between the sequence runs since samples replicated for PCR1 on the two instruments, but sequenced in Lab2.b, were highly similar. Although an error was introduced at some point in the PCR workflow, it also wasn’t attributable to PCR or extraction kit contamination based on negative controls nor a point source contaminant as several genera varied within the most affected phyla (e.g. Bacteroidota, Actinomycetota, Planctomycetota, and Candidatus Saccharibacteria).

In evaluating the overall library metrics as a diagnostic for library quality or confidence, we observed slightly more than a two-fold variation in the total number of reads (5.1–11.7 M for Lab2.b and Lab1, respectively) between sequence runs despite using similar DNA sequencing workflows. Though we were unable to definitively identify the source of this variation in read number, plausible explanations include differences in the amount of DNA loaded onto the MinION flow cell, reagent quality, and variation in the number of sequencing pores on each individual flow cell^32^. Overall, the total number of sequence reads was not a useful diagnostic as both sequence runs with the highest and lowest number of reads were considered successful based on the cross-laboratory comparisons.

An additional metric included the percentage of sequences removed at each step of the bioinformatic quality filtering pipeline and the resulting number of sequence reads per sample. Given the reportedly higher error rate of the MinION platform^33^, we chose to keep only reads with both forward and reverse barcodes present, which removed an average of 42.4% of the reads. This level of stringency would likely have removed more sequences than a single barcode requirement due to either sequencing error (i.e. barcode mismatches) or amplicon length (e.g. missing barcodes) although we did not specifically explore this option.

A useful tool in identifying an outlier sequence run was the evaluation of the number of reads removed based on quality scores after demultiplexing. The QC-quality step was effective in identifying Lab5 as an outlier with the removal of almost 10× more sequences than all other runs. The final QC-length step was not an effective diagnostic as it removed less than 0.1% of the sequences and did not differ between laboratories. The small effect of this QC step is in part due to the order of the bioinformatics pipeline as most of the erroneous sequences were already removed with the preceding steps. While we cannot be sure if the lower number of sequence reads and higher sequence removal during quality filtering in Lab5 are due to operator handling or differences in DNA sequencing chemistry (i.e. sequence kit variability, poor flow cell health, etc.), our results suggest that on average, quality filtering should only remove between 2% and 5% of the sequences following demultiplexing. Higher removal rates may warrant more scrutiny in quality assessment. We urge caution that these percentages are based on removal at each step and the percentages will change based on the order of processing or selection of criteria. Although a number of quality-filtered reads influences the final number of reads per sample as discussed below, all sequences remaining after this initial bioinformatics screening are expected to be high quality and comparable.

The simple mock community that was included as a positive control was not an effective diagnostic for a successful sequence run as all laboratories characterized the mock community at >0.85 BC similarity. In fact, the aberrant sequence run (Lab2.a) was more similar than others to the expected community. One reason the mock community failed to identify the aberrant run was incongruency in the community composition. Due to apparent taxa-specific errors introduced during PCR, the aberrant run showed significant differences in with the Actinomycetota and Bacteroidota phyla in which neither are present in the eight species ZymoBIOMICS mock community. Due to the apparent taxa-specific errors introduced in the PCR step, a mock community with similar taxonomic diversity likely would have indicated an error in the aberrant run. A potential approach toward a diagnostic would be a complex synthetic mock community that included key species from each of the most abundant phyla in the expected community. This task is daunting for commercialization and/or widespread distribution due to the impracticalities of identifying a priori the relevant taxa for diverse ecological samples and the cost associated with preparation, such as culturing, mixing, and standardization.

As demonstrated here, reference soils could potentially serve as positive controls for successful sequence runs. Comparison between replicate soils in the primary (presumably the expected) and secondary (experimental) labs by BC similarity ordination was an effective diagnostic for identifying errors from PCR (Lab2.a) and/or biases associated with library size (e.g. Lab5). This comparison identified errors for Lab5 at each workflow step and for Lab2.a in both workflow steps following PCR. Additionally, reference soils could be applied as tools to identify the overall consistency and repeatability of an experiment by using similarity indices of the observed vs expected profiles as done here.

Reference soils have been available from the United States National Institute of Standards and Technology for over 40 years. The standard methodologies for some reference soils such as air-drying and gamma radiation^34^ make them less applicable to community studies; however, publicly available or in-house soils could be used as positive controls if they are well homogenized, stored for stability, and well-characterized with multiple runs which would be best if performed by more than one laboratory. Like the simple ZymoBIOMICS mock community, publicly-available reference soil communities could exist as either genomic DNA extracts or whole soils along with a well-characterized DNA sequencing library for comparative purposes. In practice, the reference soil would be included with replication in each library preparation and used to identify overall profile similarity. We also suggest a variety of reference soils available to reflect the sample pH and texture profile, especially when included at the DNA extraction step. Extensively sequenced reference soils could also be used to help determine further potential taxonomic biases that arise from methodological and/or laboratory differences.

Across the seven sequence runs, there was a three-fold difference in the final (QC) library size. The omission of two of the three libraries by Lab3 artificially increased the number of reads per soil sample whereas the greater removal of low-quality sequences by Lab5 compared to the others resulted in samples with low reads. The two sequence runs were distinguishable from the other runs based on species richness even after rarefying all samples to a common sequencing depth. The effect of sampling depth on α- and β-diversity estimates is well known in ecological studies with diversity estimates increasing as more individuals are sampled^35^ and rarefaction (i.e. sub-sampling to a common depth) has been suggested as a potential means to remove this potential bias^36^. Due to the patchy distribution and over-dispersed nature of microbial communities a larger library size will always have more taxonomic (i.e. species, OTU, or ASV) richness as rare species will not be detected in the smaller library size. Rarefaction techniques^37^ do not differentiate between absence (zero probability of being detected) or rarity (non-zero probability of being detected but below detection level). As a result, no matter how the larger library is rarefied, the rare taxa present in that library will never be detected in the smaller library. The effect of unobserved species on α-diversity estimates following rarefaction is not a new problem^38^ and although rarefaction can improve estimates it cannot fully address the problems in estimating α-diversity, particularly in over-dispersed, incompletely sampled communities that are typical in soils. If sampling efforts are large enough, this artefact may render rarefaction inadequate to remove biases. As opposed to rarefaction, we suggest that α-diversity estimates between treatments of interest are instead compared using the relationship between species richness and sampling depth. LB plots are a potentially useful diagnostic tool to identify such differences between treatments. In this study, LB plots were successful in identifying abnormal libraries: Lab5 which had the highest removal of sequences with low-quality scores, and Lab2.a which exhibited a different community profile from the other five laboratories. Thus, LB plots are a potential tool to diagnose problematic libraries or account for the variability in library sizes when comparing similar treatments/sites in different sequence runs. In addition, LB plots may be a good tool to compare treatments when sampling depth varies within a single run.

BC index is one of the most common distance metrics used in ecology due to its sensitivity to community changes which is in part due to dependency on sample size^39^. While this may be a desired goal in individual studies where sampling efforts are often similar between treatments, the sensitivity should be considered when sampling depth differs between treatments or sequencing runs. Such problems may be particularly important in meta-analyses, cross-laboratory comparisons, or larger single studies that require multiple sequencing runs. In this study, variance partitioning showed that 47.7%, 26.6%, and 1.1% of the variance was explained by site, laboratory, and library when using BC distances. Due to the nature of the design of this study, samples produced by each laboratory differed due to sequencing depth and variation in laboratory procedures (e.g. PCR setup). As shown here, sampling depth was an important factor in variance partitioning for BC, but less so for Morisita distances although both metrics clearly identified Site and Laboratory as dominant drivers. Additionally, both metrics identified Lab2.a as an outlier in the two most autonomous libraries but only BC identified Lab5. As a result, we suggest that researchers evaluate the effect of sample size on ordination patterns and/or use distance metrics that are less sensitive to sample size to verify that treatment differences are not an artefact of sample effort.

Capturing the complexity of soils within a biological context using DNA sequencing is challenging, yet amplicon or marker-gene sequencing techniques have been widely adopted and instrumental in understanding the biological processes within the soil ecosystem. Our cross-laboratory comparison produced irregular libraries that that were not detected with common diagnostics of run statistics (e.g. total library size, quality filtering, etc.) and/or use of a simple mock community positive control. It was only through the replicated analysis by different laboratories that the irregular libraries, and more importantly an aberrant library, were identified. Additionally, variability increased with each step in the workflow in which PCR errors and differences in library size had strong impacts on measures of both α and β-diversity. We recommend the following diagnostics for library accuracy in soil microbial community analyses: (i) summary statistics of sequencing depth and sequence removal at each processing step, (ii) comparison of replicate samples for estimates of within-lab variability, and (iii) inclusion of a reference soil that can be compared across sequence runs and/or multiple laboratories. As shown by the effects of library size on α- and β-diversity estimates, regression techniques and distance indices with and without sample size bias are recommended for treatment comparisons to ensure accuracy and reproducibility in soil microbial community analyses.

## Online methods

### Study design

Six independent laboratories participated in the study by sequencing soil bacterial communities with different levels of preparation autonomy. Two laboratories (primary labs) collected soil from agricultural cropping systems typical for each region, performed soil DNA extraction, and produced a barcoded PCR library on samples from both sites. Both primary labs sent aliquots of each soil, soil DNA extract, and the barcoded PCR library to three secondary laboratories. All laboratories received aliquots of the same primer stocks.

The samples received for sequencing by the six laboratories were at varying stages of library preparation: (i) soils which required DNA extraction, PCR, and sequencing (Ext/PCR/Seq), (ii) soil DNA extracts that required only PCR and sequencing (PCR/Seq), and (iii) a DNA library prepared by the primary lab which only needed to be sequenced (Seq) (Fig. 1). All three libraries (Ext/PCR/Seq, PCR/Seq, and Seq) were pooled into a single 16S rRNA amplicon library and sequenced in a single run using the Oxford Nanopore MinION platform (Oxford Nanopore Technologies [ONT], Oxford, UK). One laboratory repeated the study bringing the total number of sequence runs to seven.

### Soils

The experiment included soils from two agricultural field experiments: the first in Fort Collins, Colorado, USA (40° 39′ 6″ N, 104° 59′ 57″ W, 1535 m elevation) and the other in Pendleton, Oregon, USA (45° 42′ N, 118° 36′ W, 438 m elevation). The Fort Collins site is located at the Colorado State University Agricultural Research and Development Education Center (ARDEC) with an average annual precipitation of 245 ± 80 mm (2013–2022) (https://coagmet.colostate.edu/) and the soil is a Fort Collins clay loam (fine-loamy, mixed, mesic Aridic Haplustalfs). The Pendleton site soil is a Walla Walla silt loam (coarse-silty, mixed, superactive, mesic Typic Haploxerolls) and the area receives approximately 413 ± 81 mm annual precipitation (1930–2018)^40^.

Soils were collected in June 2019 from four replicate plots at each site (*n* = 4). The ARDEC soils were collected from control plots of a conventionally-tilled continuous corn (*Zea mays* L.) system^41^. The Pendleton soils were sampled from plots managed in dryland annual wheat (*Triticum aestivum* L.) under no-tillage. For each site, six 2-cm diameter cores were sampled from a 0 cm to 10 cm depth either between the crop rows (ARDEC) or at the plant crowns (Pendleton). The cores were composited in a bag and stored on ice until transferred to the laboratory. Large clods were fragmented with a rolling pin. Samples were sieved to <4 mm, homogenized by hand, and aliquoted. Soils were frozen at −20 °C and shipped overnight on ice to each laboratory.

### DNA extraction and library preparation

All laboratories performed the following steps of a standard protocol starting from soil, soil DNA, and prepared libraries provided by the primary labs. From soil, DNA was extracted from 0.25 g of field moist soil in triplicate using the Qiagen DNeasy PowerSoil Pro Kit (Qiagen, Germantown, MD) per the manufacturer’s recommendations. Three soil-free extractions were included as negative kit controls (ExtH2O). Primary labs shipped aliquots of the DNA extracts in 0.65 mL Lo-Bind microcentrifuge tubes (Eppendorf, Enfield, CT) overnight on ice to secondary labs.

Soil DNA extracts generated in-house and provided by primary labs were diluted 1:20 with nuclease-free water and amplified in triplicate in 20 μL PCR reactions (PCR1) using the Bact-27F^42^ and Univ-1492R^43^ primers with ONT 5′ tail sequences (Bact-27F-Mn, 5′-TTTCTGTTGGTGCTGATATTGCAGRGTTYGATYMTGGCTCAG-3′; Univ-1492R-Mn, 5′-ACTTGCCTGTCGCTCTATCTTCTACCTTGTTACGACTT-3′; ONT tail sequences are underlined). Reactions contained 1× Phusion HSII Master Mix (Thermo Scientific, Waltham, MA), 0.2 μM forward and reverse primers, and 2 μL diluted template or water as a negative control (PCR1H2O). On average, the PCR reactions for soil samples contained 6.6 ± 1.8 ng DNA across all laboratories and both soils. The ZymoBIOMICS Microbial Community DNA Standard (product D6305; Zymo Research, Irvine, CA) was included as a positive control (MOCK) at 2 ng per reaction. The theoretical composition and relative abundance based on the 16 S rRNA gene (%) of the ZymoBIOMICS standard is *Pseudomonas aeruginosa* (4.2%), *Escherichia coli* (10.1%), *Salmonella enterica* (10.4%), *Lactobacillus fermentum* (18.4%), *Enterococcus faecalis* (9.9%), *Staphylococcus aureus* (15.5%), *Listeria monocytogenes* (14.1%), and *Bacillus subtilis* (17.4%). Amplification was performed by denaturing at 98 °C for 30 s followed by 25 cycles of a three-step reaction with 98 °C for 15 s, 50 °C for 15 s, and 72 °C for 60 s with a final extension of 72 °C for 5 min. Replicate PCR products (15 μL each) were pooled, purified with AMPure XP beads (Beckman Coulter, Indianapolis, IN) in a 1:1 volume per manufacturer’s protocol, and resuspended in 40 μL nuclease-free water. Amplification products were verified for expected size and estimated quantity using gel electrophoresis before and after the bead purification. Purified PCR1 products (including controls) were diluted 1:10 in nuclease-free water and barcoded using the PCR Barcoding Expansion 1–96 kit (ONT). Barcoding reactions were performed in 50 μL reactions with 1× Phusion HSII Master Mix, 1 μL sample-specific PCR barcode, and 5 μL diluted PCR1 product or water for the negative control (PCR2H2O). Reactions were placed in a thermocycler for the following protocol: 98 °C for 30 s followed by 15 cycles of 98 °C for 15 s, 62 °C for 15 s and 72 °C for 60 s, and a final extension at 72 °C for 5 min. Products were visualized by gel electrophoresis to confirm the addition of the barcode sequences. Barcoded products (45 μL) were purified using AMPure beads in a 1:1 ratio and resuspended in 50 μL nuclease-free water.

Barcoded PCR libraries (PCR/Seq and Ext/PCR/Seq) were individually prepared by pooling 5 μL of each reaction. The three libraries that differed in the level of preparation autonomy (Ext/PCR/Seq, PCR/Seq, Seq) were diluted to 8–10 ng μL^−1^, pooled volumetrically, and prepared for sequencing. Library preparation was initiated with 400 ng of pooled, barcoded library according to ONT 1D PCR barcoding (96) genomic DNA (SQK-LSK109) protocol (PBGE96_9068_v109_revD_23May2018) using the SQK-LSK109 Ligation Sequencing Kit and R9.4.1 (FLO-MIN106D) flow cell (ONT). Sequencing was performed for 48 h with the setting of −180 V.

Except for the sequencing device and the model of the flow cell, equipment including thermocyclers, pipet tip types (i.e. low adhesion, filtered), and centrifuges were allowed to vary based on available equipment. The lab that repeated the sequencing run (Lab2) used different thermocyclers for each experiment to evaluate the potential impact of the thermocycler model on the sequence results. For the repeated experiment (Lab2.b), a subset of samples (*n* = 4) was amplified with the two different thermocyclers for PCR1 and then treated similarly for the remaining protocol.

### Sequence processing

Basecalling and sequence processing for each of the six participating laboratories was performed by a single participating laboratory and, except when noted, default parameters were used for each processing step. To reflect the processing that typically occurs in each participating laboratory, all bioinformatics steps were conducted for each sequencing run individually. Sequences generated on the MinION platform were base-called using the high-accuracy configuration (dna_r9.4.1_450bps_hac.cfg) and demultiplexed according to both the forward and reverse barcodes (--require_barcodes_both_ends) using Guppy v6.0.1 (ONT). Sequences were filtered based on a minimum q-score of 70 using Filtlong v0.2.1^44^ followed by filtering based on length (1000–2000 bp) using Cutadapt v3.2^45^. All remaining sequences were classified using minimap2 v2.22^46^ and the default NCBI-linked Reference Database^47–49^ available from EMU v3.0.0^50^ at the bacteria species level. Following classification, EMU applies an expectation minimization algorithm to adjust taxonomic assignments using up to 50 sequence alignments per sequence read to reduce potential errors^50^. The final species-level count table generated with EMU from each sequencing run was then pooled into a single table and used in all downstream analyses.

### Statistics and reproducibility

All statistical analyses on the final pooled species-level count table were performed in R Studio v2022.07.2 using the R statistical package v4.3.1^51^. Observed species richness (Sobs) was used as the sole metric of α-diversity and calculated with the R phyloseq package v1.44.0^52^ after rarifying all samples to 12,000 reads. Differences in Sobs were tested by ANOVA for the three main effects (site, library, and sequence run nested in the library) and partial effect sizes (η^2^) were estimated with the effect size package v0.8.5^53^. Non-linear (i.e. Michaelis–Menten) regressions of the relationship between the number of original sequences read per sample and Sobs were determined for each sequence run/library combination using the R nls function in the stats v3.6.2 package. LB plots were used to compare the regressions of species richness versus the original number of sequence reads by sequence run.

A phylogenetic tree was constructed from the EMU reference database^50^ using mothur v1.42.1^54^. Briefly, sequences were aligned to the SILVA reference alignment v138 and a neighbour-joining tree was constructed with fastreeR v1.4.0^55^. The phylogenetic tree was used to develop a heatmap of the mean family-level taxonomic abundances normalized to counts per million reads. Variability in taxonomic abundances between sequence runs was calculated as the Log2FC between normalized (counts per million reads) abundances for each run relative to the base mean observed at the two primary laboratories (Lab1 and Lab4). Differences in variability (i.e. range in Log2FC across all sequence runs) were then tested using a repeated measures ANOVA with taxon as the subject and library the main effect calculated at the phylum, class, and genus taxonomic levels.

Significant differences between site, library, or sequence run were determined by perMANOVA and the data visualized by distance-based unconstrained ordinations (i.e. Principal correspondence analysis or PCoA) constructed from either BC distances of Hellinger-transformed genera abundances (i.e. counts) using the R vegan package v2.6-4^56^. Additionally, perMANOVA and PCoA were performed from Morisita distances of untransformed genera abundances as this index previously has been shown to be less sensitive to sample size^39^. The variation associated with each treatment factor (site, library, sequence run) was evaluated by variance partitioning of this same distance matrix using the adjusted *R*^2^ values derived from a distance-based redundancy analysis (db-RDA). Where appropriate the BC or Morisita distances were converted to similarities (e.g. 1 − distance). Variability was calculated as the IQR or difference between the 75th and 25th percentiles.

Differentially abundant taxa between the two sites were tested using the DESeq2 v1.40.2 package^57^ as a middle-ground approach for both Type I (false-positive) and Type II (false-negative) errors^58^. Recent research recommends the use of more conservative differential abundance tools than DESeq2 due to potential Type I error^58^; however, DESeq2 was used to evaluate variation between sequencing runs for different phyla rather than specifically contrasting the two soils which would be more affected by higher error rate^59^. For each sequence run, the differential abundance of phyla between the two soils was reported as Log2FC values. To control for differences in sample depth, all data were normalized using the relative log expression algorithm which is the default method in DESeq2. Variation in Log2FC changes at the phylum, class, and genus taxonomic levels was calculated independently for each library as the range for each taxon across all sequence runs. Differences in variability (i.e. range in Log2FC across all sequence runs) were then tested using a repeated measures ANOVA with taxon as the subject and library as the main effect.

Within-laboratory (e.g. sample-to-sample) and between-laboratory (e.g. reference soil) sample variability was evaluated as potential diagnostics. Sample variability within each sequencing run was calculated as the BC similarity between replicate samples (e.g. subsamples from the same site/plot combination) using Hellinger-transformed genera relative abundances. The potential of reference soils to identify aberrant sequencing runs was evaluated as the BC similarities of the Hellinger-transformed genera relative abundances for each individual soil sample between the secondary lab (i.e. sequence run) and the two primary labs (Supplementary Table 1).

### Reporting summary

Further information on research design is available in the Nature Portfolio Reporting Summary linked to this article.

### Supplementary information

Supplementary Information

Reporting Summary

## Data Availability

All raw sequence data for this study have been deposited in the European Nucleotide Archive (ENA) at EMBL-EBI under accession number PRJEB75585.

## Code availability

All scripts and files used for final data processing and analysis, figure generation, and phylogenetic tree construction are available on GitHub (https://github.com/DanielManter-USDA/16S_CLC), as well as a Zenodo repository^60^.
