# Supplementary material for: Unveiling errors in soil microbial community sequencing: a case for reference soils and improved diagnostics for nanopore sequencing
Source: Commun Biol. 2024 Jul 28;7:913. doi: 10.1038/s42003-024-06594-8 (PMC11284219; doi:10.1038/s42003-024-06594-8)

Supplementary Information

Unveiling errors in soil microbial community sequencing: A case for reference soils and improved diagnostics for nanopore sequencing

Daniel K. Manter[1*], Catherine L. Reardon[2*], Amanda J. Ashworth[3], Abasiofiok M. Ibekwe[4], R. Michael Lehman[5], Jude E. Maul[6], Daniel N. Miller[7], Timothy Creed[1], Patrick M. Ewing[8], Stanley Park[4], Thomas F. Ducey[9], Heather L. Tyler[10], Kristen S. Veum[11], Sharon L. Weyers[12], David B. Knaebel[13]

[1] Soil Management and Sugar Beet Research, United States Department of Agriculture, Agricultural Research Service (USDA-ARS), Fort Collins, CO, 80526, USA
[2]Soil and Water Conservation Research Unit, USDA-ARS, Adams, OR, 97810, USA
[3]Poultry Production and Product Safety Research Unit, USDA-ARS, Fayetteville, AR 72701, USA
[4]Water Efficiency and Salinity Research Unit, USDA-ARS, Riverside, CA, 92507, USA
[5]North Central Agricultural Research Laboratory, USDA-ARS, Brookings, SD, 57006, USA
[6]Sustainable Agricultural Systems Laboratory, USDA-ARS, Beltsville, MD, 20705, USA
[7]Agroecosystem Management Research Unit, USDA-ARS, Lincoln, NE, 68583, USA
[8]Food Systems Research Unit, Burlington, VT, 05405, USA
[9]Coastal Plains Soil, Water and Plant Research Center, USDA-ARS, Florence, SC, 29501, USA
[10]Crop Production Systems Research Unit, USDA-ARS, Stoneville, MS, 38776, USA
[11]Cropping Systems and Water Quality Research Unit, USDA-ARS, Columbia, MO, 65211, USA
[12]Soil Management Research Unit, USDA-ARS, Morris, MN, 56267, USA
[13]Federal government retiree, Fayetteville, NY, 13066, USA

[*]These authors contributed equally to this work
[*]Corresponding authors: daniel.manter@usda.gov, catherine.reardon@usda.gov

Supplementary Table 1. Sequence reads for each independent laboratory sequencing run.

Sequence Run[†]	Primary Lab	Total Reads	Step 1: De-multiplexed[‡]	Step 2: QC-quality[‡]	Step 3: QC-length[‡]	Reads per sample[§]				
						Soil	MOCK	ExtH2O	PCR1H2O	PCR2H2O
Lab1	Lab4	11,668,288	6,007,575 (48.5%)	5,660,861 (3.0%)	5,652,208 (0.1%)	73,116	113,493	4,166	3,299	0
Lab2.a	Lab4	6,744,159	3,987,512 (40.9%)	3,836,024 (2.2%)	3,832,081 (0.1%)	49,971	77,986	22	16	0
Lab2.b	Lab4	5,113,254	2,930,315 (42.7%)	2,809,575 (2.4%)	2,806,522 (0.1%)	34,208	54,508	121	45	0
Lab3	Lab4	8,475,382	4,922,056 (41.9%)	4,518,245 (4.8%)	4,509,075 (0.1%)	173,298	223,206	35,905	19,014	0
Lab4	Lab1	8,338,972	4,972,624 (40.4%)	4,463,900 (6.1%)	4,394,347 (0.8%)	57,846	69,731	1,715	1,604	0
Lab5	Lab1	5,366,425	3,106,774 (42.1%)	1,798,667 (24.4%)	1,796,529 (0.0%)	22,877	29,707	4,177	7,570	0
Lab6	Lab1	10,720,254	6,368,833 (40.6%)	5,836,426 (5.0%)	5,825,081 (0.1%)	75,281	122,365	2,517	5,033	2

[†] The secondary laboratory, Lab2, repeated the experiment and generated two sequence runs (Lab2.a and Lab2.b). Lab3 sequenced only one of the three libraries.

[‡] Values indicate reads remaining and the numbers in parentheses are the percent of reads removed compared to the prior step.

[§] MOCK, the ZymoBIOMICS mock DNA standard, was included as a positive control. Negative controls include soil-free DNA extractions (ExtH2O, n=3), gene-specific PCR with no template (PCR1H2O, n=1), and the barcoding PCR with water template (PCR2H2O, n=1).

Supplementary Figure 1. Relative abundance of genera in the MOCK sample. Data is for the Ext/PCR/Seq library only. Numbers above the bars are Bray-Curtis similarity between the expected ZymoBIOMICS Microbial Community DNA Standard (Expected) and laboratory-generated (MOCK) data.

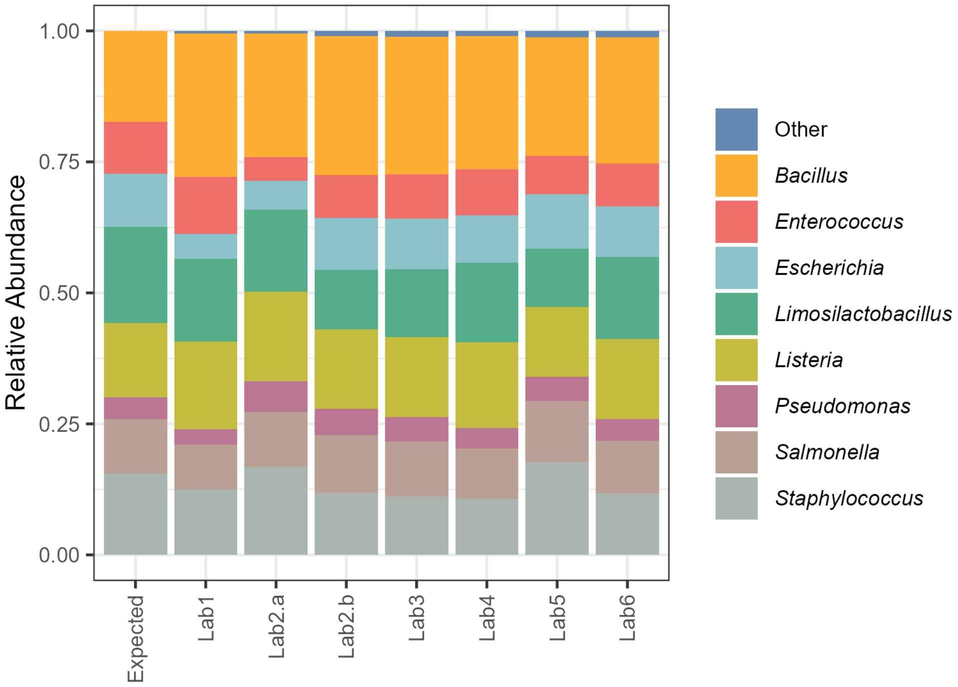

Supplementary Figure 2. Principal correspondence analysis (PCoA) of Bray-Curtis distances and relationship of PCoA axes to sequence reads. Data include two soil sites analyzed by six participating laboratories with increasing levels of process autonomy (Seq < PCR/Seq < Ext/PCR/Seq). Lab2 repeated the experiment and is shown as Lab2.a (first run) and Lab2.b (repeat). A) PCoA was constructed from Bray-Curtis distances calculated from Hellinger-transformed genera abundance data. B) Relationship between PCoA axes (Axis 1 = top panel, Axis 2 = bottom panel) and sequence reads.

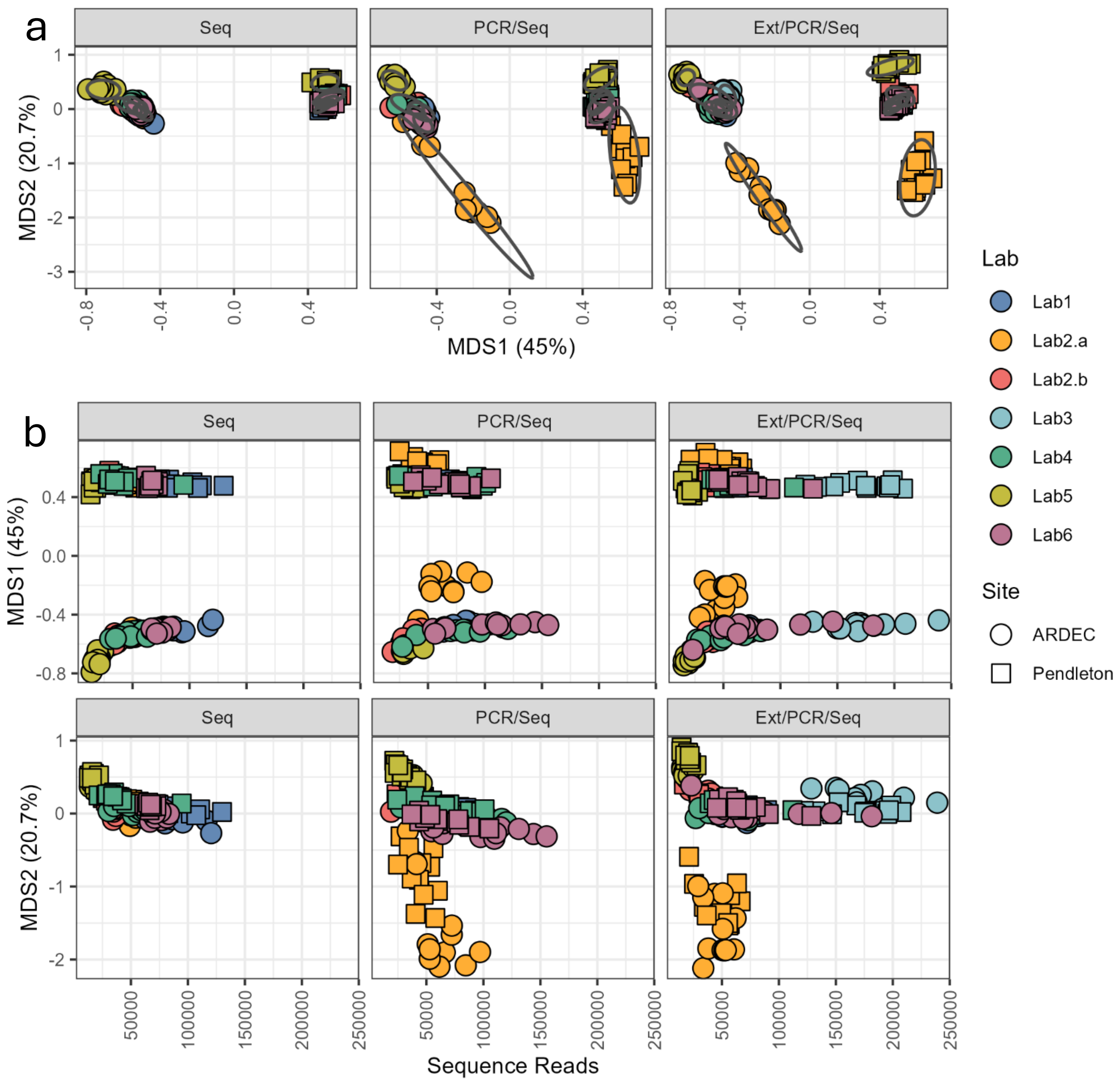

Supplementary Figure 3. Principal correspondence analysis (PCoA) of Morisita distances and relationship of PCoA axes to sequence reads. Data include two soil sites analyzed by six participating laboratories with increasing levels of process autonomy (Seq < PCR/Seq < Ext/PCR/Seq). Lab2 repeated the experiment and is shown as Lab2.a (first run) and Lab2.b (repeat). A) PCoA was constructed from Morisita distances calculated from untransformed genera abundance data. B) Relationship between PCoA axes (Axis 1 = top panel, Axis 2 = bottom panel) and sequence reads.

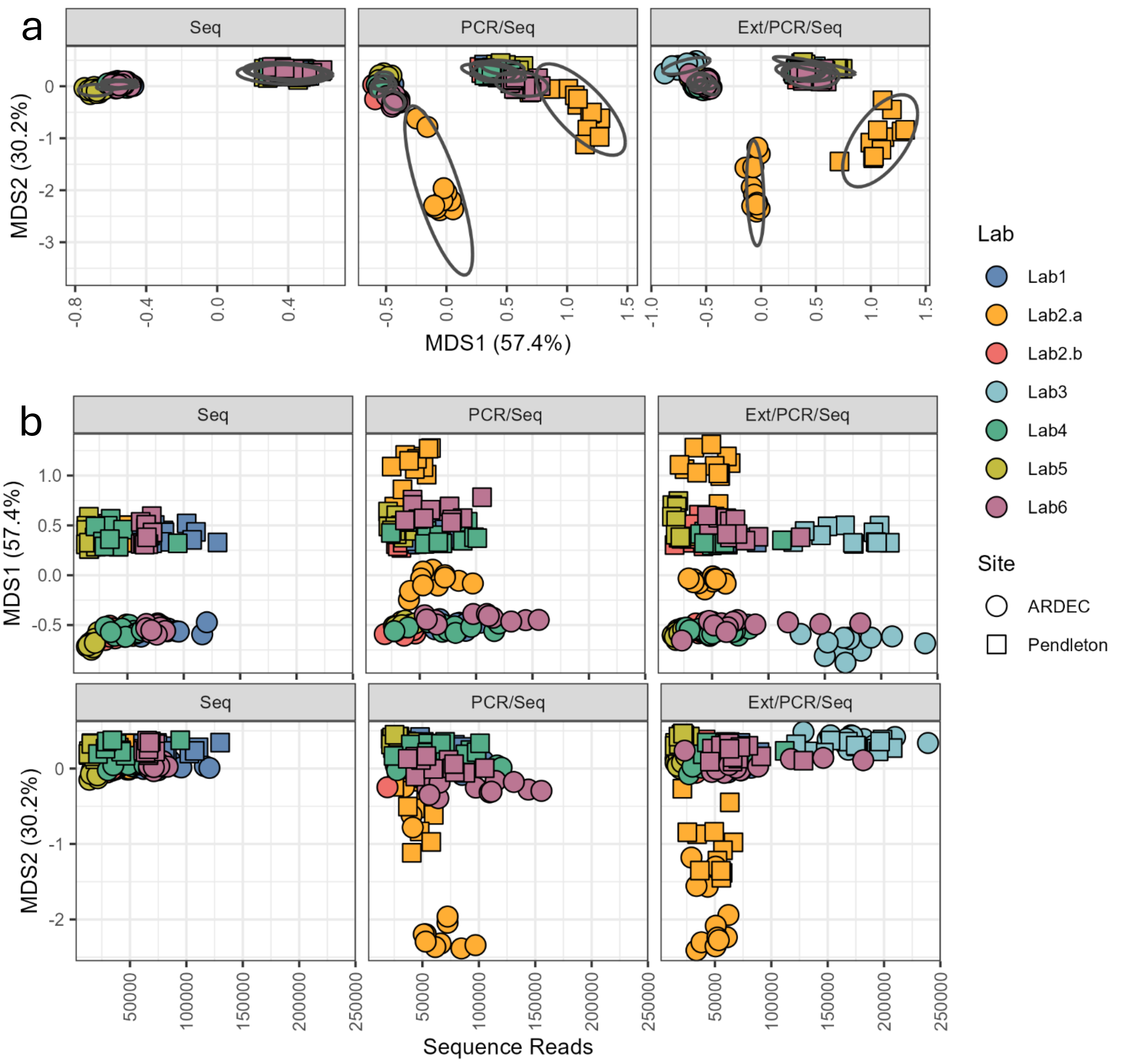

Supplement: Supplementary file 1 — Supplementary Information [file 42003_2024_6594_MOESM1_ESM.pdf]
