# Supplementary material for: Unveiling errors in soil microbial community sequencing: a case for reference soils and improved diagnostics for nanopore sequencing
Source: Commun Biol. 2024 Jul 28;7:913. doi: 10.1038/s42003-024-06594-8 (PMC11284219; doi:10.1038/s42003-024-06594-8)
Supplement: Supplementary file 2 — Reporting Summary [file 42003_2024_6594_MOESM2_ESM.pdf]

nature portfolio

Corresponding author(s): Daniel K. Manter
Catherine L. Reardon

Last updated by author(s): 6/4/2024

Reporting Summary

Nature Portfolio wishes to improve the reproducibility of the work that we publish. This form provides structure for consistency and transparency in reporting. For further information on Nature Portfolio policies, see our Editorial Policies and the Editorial Policy Checklist.

Statistics

For all statistical analyses, confirm that the following items are present in the figure legend, table legend, main text, or Methods section.

n/a	Confirmed	
☐	☒	The exact sample size (*n*) for each experimental group/condition, given as a discrete number and unit of measurement
☐	☒	A statement on whether measurements were taken from distinct samples or whether the same sample was measured repeatedly
☐	☒	The statistical test(s) used AND whether they are one- or two-sided<br>*Only common tests should be described solely by name; describe more complex techniques in the Methods section.*
☐	☒	A description of all covariates tested
☐	☒	A description of any assumptions or corrections, such as tests of normality and adjustment for multiple comparisons
☐	☒	A full description of the statistical parameters including central tendency (e.g. means) or other basic estimates (e.g. regression coefficient) AND variation (e.g. standard deviation) or associated estimates of uncertainty (e.g. confidence intervals)
☐	☒	For null hypothesis testing, the test statistic (e.g. *F*, *t*, *r*) with confidence intervals, effect sizes, degrees of freedom and *P* value noted<br>*Give P values as exact values whenever suitable.*
☐	☒	For Bayesian analysis, information on the choice of priors and Markov chain Monte Carlo settings
☐	☒	For hierarchical and complex designs, identification of the appropriate level for tests and full reporting of outcomes
☐	☒	Estimates of effect sizes (e.g. Cohen's *d*, Pearson's *r*), indicating how they were calculated

Our web collection on statistics for biologists contains articles on many of the points above.

Software and code

Policy information about availability of computer code

Data collection	Software: Guppy v6.0.1, Filtlong v0.2.1, Cutadapt v3.2, minimap2 v2.22, mothur v1.42.1, EMU v3.0.0, R Studio v2022.07.02, and R v4.3.1. R packages: phyloseq v1.44.0, effectsize v0.8.5, stats v3.6.2, fastreeR v1.4.0, vegan v2.6-4, DESeq2 v1.40.2
Data analysis	All code and raw data are available in a GitHub (https://github.com/DanielManter-USDA/16S_CLC) as well as a Zenodo repository (https://doi.org/10.5281/zenodo.11557861).

For manuscripts utilizing custom algorithms or software that are central to the research but not yet described in published literature, software must be made available to editors and reviewers. We strongly encourage code deposition in a community repository (e.g. GitHub). See the Nature Portfolio guidelines for submitting code & software for further information.

Data

Policy information about availability of data

All manuscripts must include a data availability statement. This statement should provide the following information, where applicable:

- Accession codes, unique identifiers, or web links for publicly available datasets
- A description of any restrictions on data availability
- For clinical datasets or third party data, please ensure that the statement adheres to our policy

All code and raw data are available in a GitHub (https://github.com/DanielManter-USDA/16S_CLC) as well as a Zenodo repository (https://doi.org/10.5281/zenodo.11557861).

Research involving human participants, their data, or biological material

Policy information about studies with human participants or human data. See also policy information about sex, gender (identity/presentation), and sexual orientation and race, ethnicity and racism.

Reporting on sex and gender	NA
Reporting on race, ethnicity, or other socially relevant groupings	NA
Population characteristics	NA
Recruitment	NA
Ethics oversight	NA

Note that full information on the approval of the study protocol must also be provided in the manuscript.

Field-specific reporting

Please select the one below that is the best fit for your research. If you are not sure, read the appropriate sections before making your selection.

☐ Life sciences ☐ Behavioural & social sciences ☒ Ecological, evolutionary & environmental sciences

For a reference copy of the document with all sections, see nature.com/documents/nr-reporting-summary-flat.pdf

Ecological, evolutionary & environmental sciences study design

All studies must disclose on these points even when the disclosure is negative.

Study description	Six independent laboratories participated in the study by sequencing soil bacterial communities with different levels of preparation autonomy. Two laboratories (primary labs) collected soil from agricultural cropping systems typical for each region, performed soil DNA extraction, and produced a barcoded PCR library on samples from both sites. Both primary labs sent aliquots of each soil, soil DNA extract, and the barcoded PCR library to three secondary laboratories. All laboratories received aliquots of the same primer stocks.<br><br>The samples received for sequencing by the six laboratories were at varying stages of library preparation: (i) soils which required DNA extraction, PCR, and sequencing (Ext/PCR/Seq), (ii) soil DNA extracts that required only PCR and sequencing (PCR/Seq), and (iii) a DNA library prepared by the primary lab which only needed to be sequenced (Seq) (Fig. 1). All three libraries (Ext/PCR/Seq, PCR/Seq, Seq) were pooled into a single 16S rRNA amplicon library and sequenced in a single run using the Oxford Nanopore MinION platform (Oxford Nanopore Technologies [ONT], Oxford, UK). One laboratory repeated the study bringing the total number of sequence runs to seven.
Research sample	Soil samples from two conventional agricultural sites.
Sampling strategy	Soils were collected in June 2019 from four replicate plots at each site (n = 4). The ARDEC soils were collected from control plots of a conventionally-tilled continuous corn (Zea mays L.) system as previously described40. The Pendleton soils were sampled from plots managed in dryland annual wheat (Triticum aestivum L.) under no-tillage. For each site, six 2-cm diameter cores were sampled from a 0-10 cm depth either between the crop rows (ARDEC) or at the plant crowns (Pendleton). The cores were composited in a bag and stored on ice until transfer to the laboratory. Large clods were fragmented with a rolling pin. Samples were sieved to <4 mm, homogenized by hand, and aliquoted. Soils were frozen at -20°C and shipped overnight on ice to each laboratory.
Data collection	All participating laboratories were responsible for data collection and DNA sequencing.
Timing and spatial scale	Sample collection occurred in June 2019.
Data exclusions	No data was excluded.
Reproducibility	All attempts to collect the data and its reproducibility are reported in the manuscript.
Randomization	Each laboratory was responsible for randomization of samples to individual wells of the PCR and sequencing steps.
Blinding	All laboratories received the samples without identifying information and only during the data analysis steps was this information added.

Did the study involve field work? ☒ Yes ☐ No

Field work, collection and transport

Field conditions	Both field sites used in this study are part of longterm research projects conducted by local USDA-ARS and university partners.
Location	The experiment included soils from two agricultural field experiments: the first in Fort Collins, Colorado, USA (40° 39′ 6″ N, 104° 59′ 57″ W, 1535 m elevation) and the other in Pendleton, Oregon, USA (45° 42′ N, 118° 36′ W, 438 m elevation). The Fort Collins site is located at the Colorado State University Agricultural Research and Development Education Center (ARDEC) with average annual precipitation of 245 ± 80 mm (2013-2022) (https://coagmet.colostate.edu/) and the soil is a Fort Collins clay loam (fine-loamy, mixed, mesic Aridic Haplustalfs). The Pendleton site soil is a Walla Walla silt loam (coarse-silty, mixed, superactive, mesic Typic Haploxerolls) and the area receives approximately 413 ± 81 mm annual precipitation (1930-2018). The ARDEC soils were collected from control plots of a conventionally-tilled continuous corn (Zea mays L.) system as previously described. The Pendleton soils were sampled from plots managed in dryland annual wheat (Triticum aestivum L.) under no-tillage.
Access & import/export	No permits were required for this research.
Disturbance	Minimal disturbance occurred at each site and consisted of six 2-cm diameter cores were sampled from a 0-10 cm depth either between the crop rows (ARDEC) or at the plant crowns (Pendleton).

Reporting for specific materials, systems and methods

We require information from authors about some types of materials, experimental systems and methods used in many studies. Here, indicate whether each material, system or method listed is relevant to your study. If you are not sure if a list item applies to your research, read the appropriate section before selecting a response.

Materials & experimental systems

n/a	Involved in the study
☒	☐ Antibodies
☒	☐ Eukaryotic cell lines
☒	☐ Palaeontology and archaeology
☒	☐ Animals and other organisms
☒	☐ Clinical data
☒	☐ Dual use research of concern
☒	☐ Plants

Methods

n/a	Involved in the study
☒	☐ ChIP-seq
☒	☐ Flow cytometry
☒	☐ MRI-based neuroimaging

Plants

Seed stocks	NA
Novel plant genotypes	NA
Authentication	NA
